# Identification of novel susceptibility loci associated with hepatitis B surface antigen seroclearance in chronic hepatitis B

**DOI:** 10.1371/journal.pone.0199094

**Published:** 2018-07-05

**Authors:** Tae Hyung Kim, Eun-Ju Lee, Ji-Hye Choi, Sun Young Yim, Sunwon Lee, Jaewoo Kang, Yoo Ra Lee, Han Ah Lee, Hyuk Soon Choi, Eun Sun Kim, Bora Keum, Yeon Seok Seo, Hyung Joon Yim, Yoon Tae Jeen, Hoon Jai Chun, Hong Sik Lee, Chang Duck Kim, Hyun Goo Woo, Soon Ho Um

**Affiliations:** 1 Department of Internal Medicine, Korea University College of Medicine, Seoul, Korea; 2 Department of Physiology, Ajou University School of Medicine, Suwon, Korea; 3 Department of Biomedical Science, Graduate School, Ajou University, Suwon, Korea; 4 Department of Computer Science and Engineering, Korea University College of Informatics, Seoul, Korea; Universita degli Studi di Pisa, ITALY

## Abstract

**Background/Aims:**

The seroclearance of hepatitis B virus (HBV) surface antigen (HBsAg) is regarded as a functional cure of chronic hepatitis B (CHB) although it occurs rarely. Recently, several genome-wide association studies (GWASs) revealed various genetic alterations related to the clinical course of HBV infection. However, all of these studies focused on the progression of HBV infection to chronicity and had limited application because of the heterogeneity of HBV genotypes. In the present study, we aimed to determine susceptibility genetic markers for seroclearance of HBsAg in CHB patients with a homogenous viral genotype.

**Methods:**

One hundred patients with CHB who had experienced HBsAg seroclearance before 60 years of age and another 100 with CHB showing high serum levels of HBsAg even after 60 years of age were enrolled. Extreme-phenotype GWAS was conducted using blood samples of participants.

**Results:**

We identified three single nucleotide polymorphisms, rs7944135 (*P* = 4.17 × 10^−6^, odds ratio [OR] = 4.16, 95% confidence interval [CI] = 2.27–7.63) at 11q12.1, rs171941 (*P* = 3.52×10^−6^, OR = 3.69, 95% CI = 2.13–6.42) at 5q14.1, and rs6462008 (*P* = 3.40×10^−6^, OR = 0.34, 95% CI = 0.22–0.54) at 7p15.2 as novel susceptibility loci associated with HBsAg seroclearance in patients with CHB. The flanking genes at these loci including *MPEG1*, *DTX4*, *MTX3*, and *HOXA13* were suggested to have functional significance. In addition, through functional analysis, *CXCL13* was also presumed to be related.

**Conclusions:**

To the best of our knowledge, this study is the first GWAS regarding the seroclearance of HBsAg in CHB patients. We identify new susceptibility loci for cure of CHB, providing new insights into its pathophysiology.

## Introduction

Approximately 686,000 people die each year due to severe and advanced liver diseases such as cirrhosis and cancer induced by chronic hepatitis B (CHB) infection [1–3]. In Korea, public vaccination programs have decreased the incidence of hepatitis B virus (HBV) surface antigen (HBsAg) seropositive patients, but the incidence remains as high as 3% of the entire population because of higher carrier rates among the people over 30 years old who did not undergo universal vaccination and presumed to have been at risk of perinatal (vertical) transmission and horizontal transmission in early childhood [4].

Clinically, the seroclearance of HBsAg is one of the most important goals in the treatment of CHB and is regarded as a safe marker for the discontinuation of antiviral treatment with nucleos(t)ide analogues. HBsAg seroclearance also has a better prognosis than a persistent HBsAg seropositive state [5,6]. Thus, HBsAg seroclearance has been recognized as a marker for permanent remission from HBV infection if there is no pre-existing cirrhosis or viral superinfection and designated as a functional cure in current practice guidelines [7]. However, the rate of HBsAg seroclearance among patients with CHB during clinical management is extremely low worldwide (0.7–1.9% per year)[8–12] and much lower in Korea (0.4% per year)[13], possibly because of differences in prevalent HBV genotype or transmission age [13,14].

Previously, potential clinical variables associated with HBsAg seroclearance in patients with CHB have been suggested, including old age and a low viral load, as determined by low serum HBV DNA or HBsAg levels [11,15–18]. However, these findings appear to just reflect the natural corollary of rapid viral elimination in patients prone to attain earlier seroclearance of HBsAg. Meanwhile, recent genome-wide association studies (GWASs) have identified multiple genomic loci associated with the risk of chronic HBV infection, including human leukocyte antigen (HLA) loci and non-HLA loci [19–21]. However, the findings of previous GWASs require careful interpretation because most of these studies involved subjects with an uncertain history of HBV exposure or an unidentified age of infection [21–24]. The age of the subject at the time of infection is the most important factor that determines the natural course following HBV infection [25]. The majority of the HBsAg negative subjects in previous studies probably had recovered from acute HBV infections during adulthood, whereas the HBsAg carriers recruited were most likely to have been infected with HBV during the perinatal period or preschool age since the studies were performed in endemic areas of HBV. Accordingly, it is uncertain whether the genetic markers disclosed in previous GWASs reflect the genetic mechanisms involved in chronicization of acute HBV infection or those involved in remission of long-term chronic HBV infection. Considering this ambiguity in previous studies, in the present study, we aimed to search for relevant genetic factors focusing on viral clearance in CHB patients. Accordingly, only subjects who had a well-documented history of chronic HBV infection were included and those who had undergone spontaneous remission from an acute HBV infection were excluded from the study.

Clinical studies have indicated that an older age (≥ 60 vs. 30–39 years old, *P* = 0.007, odds ratio [OR] = 6.07) and a lower HBsAg titer (< 10 vs. ≥ 1000 IU/mL, *P* <0.001, OR = 13.2) are significantly associated with HBsAg seroclearance in patients with CHB [15,17]. In this study, therefore, an extreme-phenotype GWAS analysis was performed by exploring single nucleotide polymorphisms between patients with CHB who achieved HBsAg seroclearance before 60 years of age and those who exhibited very high serum levels of HBsAg (≥1000 IU/mL) even after the age of 60 years, having low possibility of HBsAg seroclearance in the future. The present study aimed to identify novel associated loci that might expand our knowledge of the genetic mechanism of HBsAg seroclearance in patients with CHB.

## Materials and methods

### Subjects and ethics

A total of 200 patients who had visited the liver clinic of Korea University Anam Hospital for the follow-up of CHB and who fulfilled the inclusion criteria were enrolled prospectively and consecutively from January 2014 to January 2016. All the patients had been diagnosed as CHB and were regularly followed-up between 2000 and 2014 in our clinic at least twice a year (every 6 months) with routine laboratory tests for serum biochemistry and/or HBV virus markers. Half of them (*n* = 100) corresponded to the case group and another half (*n* = 100) to the control group. The patient group was defined as follows: the case group included patients who had experienced HBsAg seroclearance at < 60 years of age and the control group included those who had exhibited a high level (> 1000 IU/mL) of HBsAg at ≥ 60 years of age. All of the study subjects were of Korean ethnicity. Diagnosis of chronic HBV infection was established based on the seropositivity of HBsAg over a 6-month period. The family history of CHB was confirmed through an inquiry or record query process. The seroclearance of HBsAg was defined as the HBsAg level measuring less than the detection limit (0.05 IU/mL), as obtained twice consecutively at least one year apart by the Architect HBsAg QT assay (Abbott Laboratories, Chicago, IL, USA). It was also defined as sero-negative in qualitative HBsAg and anti-HBs assay by commercial methods (COBAS e601, Roche diagnostics, Indianapolis, IN, USA). HBV DNA was measured by COBAS TaqMan® real-time PCR quantification method (Roche Diagnostics) with a detection limit of 20 IU/ml. Hepatitis B e antigen (HBeAg) and anti-HBe were confirmed by immunoradiometric assay (North Institute of Biological Technology, Beijing, China), was used according to the manufacturer’s instruction. None of the participants presented any evidence of concomitant hepatitis C virus, hepatitis D virus, or human immunodeficiency virus infection. All the participants provided written informed consent for participation, the use of their medical data and collection of serum samples for research purpose. This project was approved by the ethics committees at Korea University Anam Hospital (ED13220) and conducted in agreement with the ethical principles of the Declaration of Helsinki.

### Single-nucleotide polymorphism (SNP) genotyping

Two hundred samples were genotyped using a HumanOmni2.5–8 BeadChip (Illumina Inc., San Diego, CA, USA), which includes 2,372,784 SNP loci. Samples were processed according to the Illumina Infinium-II assay manual. SNP quality control for all sets of samples was applied as follows: SNPs were excluded if they (i) were not located on autosomal chromosomes, or had (ii) a call rate of <0.95 in both cases and controls, (iii) a minor allele frequency of <0.01, or (iv) significant deviation from the Hardy–Weinberg equilibrium of (P < 1.0 × 10^−5^) in controls. Similarly, all the samples were identified with a genotyping rate > 96% and a potential genetic relatedness based on pair–wise identity by state. The remaining 1,365,088 SNPs were used in subsequent analyses for association.

### GWAS

The associations between genotypes and chronic HBV clearance were assessed in an additive model by logistic regression with covariate adjustment for sex using PLINK v 1.07 (http://zzz.bwh.harvard.edu/plink). The quantile–quantile plot was generated using R (3.3.2) (http://www.r-project.org/) to evaluate the overall significance of the genome-wide associations and the potential impact of population stratification. The impact of population stratification was also evaluated by calculating the genomic control inflation factor.

The proxy SNPs with regional recombination rates were assessed using LocusZoom (http://locuszoom.org/) and the 1000 Genomes Project data. Haplotype blocks were generated from the Haploview software (http://www.broadinstitute.org/haploview). A haplotype association test was performed using the hap-logistic option in PLINK. A binomial multivariate logistic regression analysis was performed with using R. *P*-values ≤ 0.05 were considered significant.

Gene ontology analysis was performed on the gene sets harboring the identified SNPs, using DAVID (https://david.ncifcrf.gov). The pathway analysis was performed on the gene sets harboring the identified SNPs, using i-GSEA4GWAS-v2 [26]. Regarding the other options, default values preset in DAVID and i-GSEA4GWAS were employed.

## Results

### Clinical characteristics of participants

To determine novel loci conferring susceptibility to functional cure from chronic HBV infection, a primary GWAS screen was performed with the aforementioned 200 chronic carriers of HBsAg, who were divided into case (*n* = 100) and control (*n* = 100) groups according to the absence or a high level of HBsAg in serum at ≥ 60 years of age. The clinical characteristics of the two groups are summarized in Table 1. The case group was younger than the control group at the time of initial diagnosis of CHB, the initial visit to our clinic, and when GWAS was performed (mean age 60.6 ± 9.3 vs. 69.3 ± 5.4 years, *P* <0.001), while showing longer duration of total follow-up. The case group included significantly more male subjects (71%) than the control group (45%) (odds ratio [OR] = 0.37, *P* <0.001). At initial visit, the subjects in the control group exhibited higher levels of serum aminotransferase and were mostly seropositive for HBeAg compared to those in the case group. Thus, more number of patients received antiviral treatment in the control group than in the case group (84% vs 31%, *P* <0.001), using oral nucleos(t)ide analogues or interferon (IFN), or both. The duration of oral nucleos(t)ide analogue treatment did not differ between the control and case groups (7.3±3.7 vs 7.7±4.0 years). However, at the time of GWAS, 13 subjects in the control group were still seropositive for HBeAg in spite of their old age, while all the subjects in the case group were seronegative for HBeAg, and 66 of them showed anti-HBs seropositivity. The familial history of chronic HBV infection in the mother or siblings was obvious in 151 of 200 patients with the comparable incidences between the case and control groups (70% vs 81%); only 3 insisted on the definite absence of CHB in their family, and for the remaining patients, familial history was uncertain. The comparison of clinical features between the subjects with and without confirmed family history are shown in S1 Table.

**Table 1:** Clinical features of participants in the present study.

		Case (n = 100) HBsAg (-) at 60 years old	Control (n = 100) HBsAg >1000IU/mL at 60 years old	*P*-value
**Male, *n***		71	45	< 0.001^a^
**Age at initial diagnosis, years**		36.0±11.3	47.0±13.0	< 0.001^b^
**Age at 1**^**st**^ **visit, years**		43.9±11.4	59.9±7.7	< 0.001^b^
**Age at GWAS, years**		60.6±9.3	69.3±5.4	<0.001^b^
**Follow-up duration, years**		16.7±8.1	9.5±5.8	<0.001^b^
**Age at HBsAg seroclearance, years**		54.7±8.9	-	-
**ALT at 1**^**st**^ **visit, IU/L**		36.0 (17–73)	49 (25–104)	0.032^c^
**ALT at GWAS, IU/L**		18.0 (13–27)	22.0 (17–29)	0.012^c^
**HBeAg (+) at 1**^**st**^ **visit, *n***		30	52	0.002^a^
**HBeAg (+) at GWAS, *n***		0	13	-
**Anti-HBe (+) at 1**^**st**^ **visit, *n***		70	42	< 0.001^a^
**Anti-HBe (+) at GWAS, *n***		90	60	< 0.001^a^
**HBsAg (+) at 1**^**st**^ **visit, *n***		100	100	-
**HBsAg (+) at GWAS, *n***		0	100	-
**Anti-HBs (+) at GWAS, *n***		66	0	0.218^a^
**HBV DNA < 20 IU/mL at GWAS, *n***		94	75	<0.001^a^
**CHB in mother or siblings, *n***				
**Present**		70	81	0.071
**Absent**		3	0	
**Uncertain**		27	19	
**Treatment type:**	**Non-treated, *n***	69	16	< 0.001^a^
	**IFN only, *n***	7	0	
	**NA only, *n***	22	81	
	**IFN & NA, *n***	2	3	
**Age of treatment start, years**		43.1±11.6	61.3±6.6	<0.001^b^
**NA treatment period, years**		7.3±3.7	7.7±4.0	0.639^b^

Data presented as number, mean ± standard deviation, or median (IQR). CHB, chronic hepatitis B; GWAS, genome-wide association study; ALT, alanine aminotransferase (normal range 5–40 U/L); HBeAg, hepatitis B e antigen; IFN, interferon (including PEGylated-interferon); NA, nucleos(t)ide analogues.

a, Chi- square test

b, Student's t-test

c, Mann-Whitney U test.

### GWAS profiling of patients with CHB showing the presence or absence of HBsAg seroclearance

**Fig 1:**
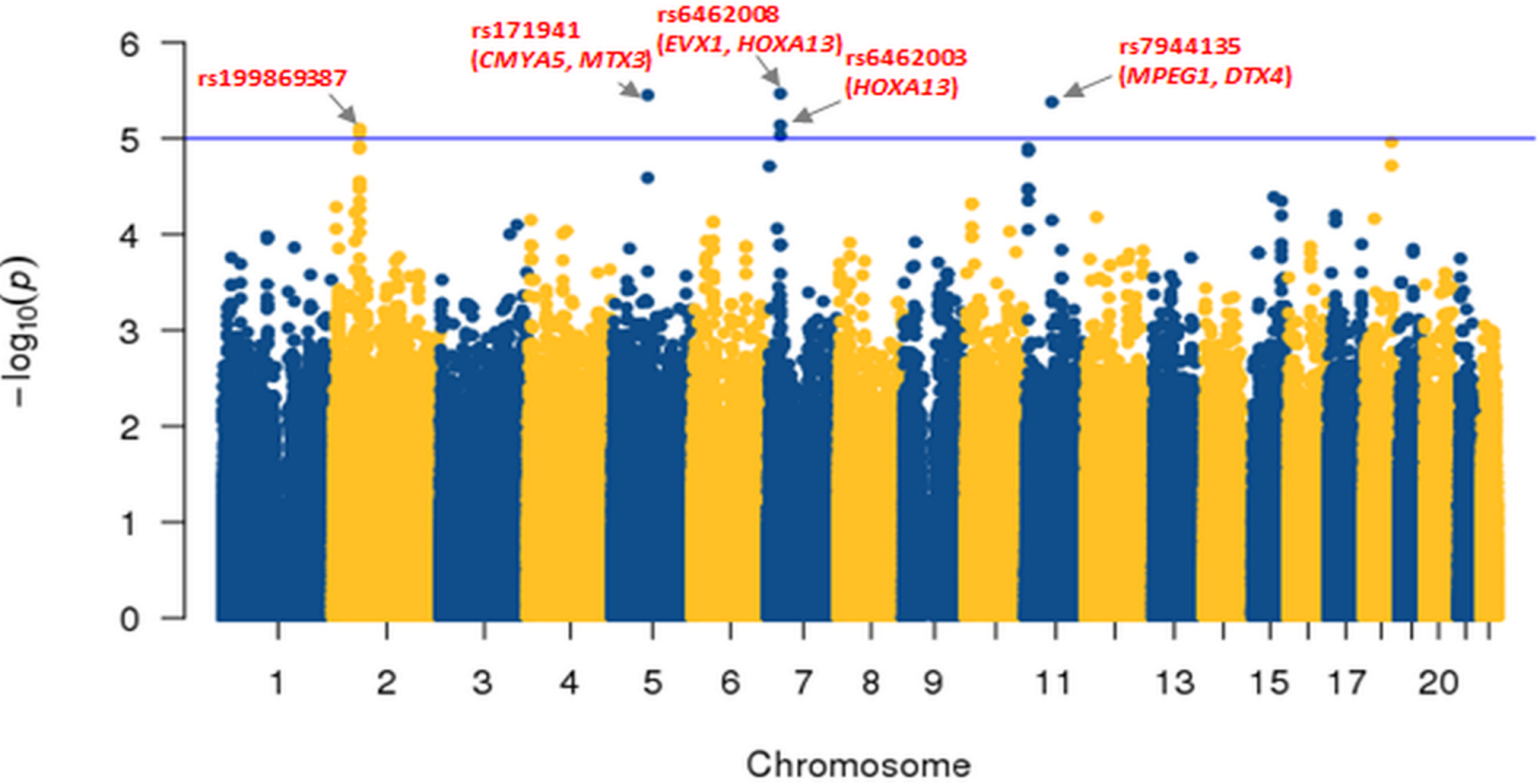
Chromosomal distribution of the single-nucleotide polymorphisms (SNPs) associated with the seroclearance of hepatitis B surface antigen (HBsAg) in patients with chronic hepatitis B (CHB). The Manhattan plot shows the chromosomal distribution of the–log10 (*P* value) of SNPs. The threshold cutoff for the significant SNPs (*P* <10^−5^) is shown with horizontal line.

Logistic association analyses were conducted between the SNPs and the seroclearance of HBsAg in patients with CHB. Quantile–quantile plot analysis showed a low genomic inflation factor (λ = 0.893), indicating that the association results were not influenced by population stratification (S1 Fig). Of the 1,365,088 SNP probes, we identified 52 SNPs associated with the status of HBsAg seroclearance with cutoff *P*-value of 10^−4^, correcting the effects of gender (S2 Table). The three SNPs (rs6462008, rs171941, and rs7944135) were found as the most significant markers (OR = 0.34, 3.69, and 4.16; *P* = 3.40×10–6, 3.52×10–6, and 4.17×10–6, respectively), and reside at chromosomes 7p15.2, 5q14.1, and 11q12.1, respectively (Fig 1).

Next, to address the effects of the allelic changes of SNPs on gene functions, we examined the flanking genes residing at the regions within 150 kb up- and down-stream from each significantly associated SNP locus. The rs6462008 is located near even-skipped homeobox 1 (*EVX1*) and homeobox A13 (*HOXA13*) on chromosome 7p15.2 (*P* = 3.40×10–6, OR = 0.34, 95% CI = 0.37–0.42). The rs171941 is located near cardiomyopathy associated 5 (*CMYA5*) and metaxin 3 (*MTX3*) on chromosome 5q14.1 (*P* = 3.52×10–6, OR = 3.69, 95% CI = 2.13–6.42). The rs7944135 is located near the macrophage expressed 1 (*MPEG1*) and deltex E3 ubiquitin ligase 4 (*DTX4*) on chromosome 11q12.1 (*P* = 4.17×10–6, OR = 4.16, 95% CI = 2.27–7.63).

In addition, we examined SNPs previously reported to be associated with chronic HBV infection. Twenty-four SNPs were obtained from the literatures and examined. Most of those SNPs were not significant in our data set (S3 Table). Only rs1419881 and rs10484569 showed marginal significance (*P* = 0.024 and 0.035).

### Identification of the regions of interest (ROIs) for the significant SNPs

**Fig 2:**
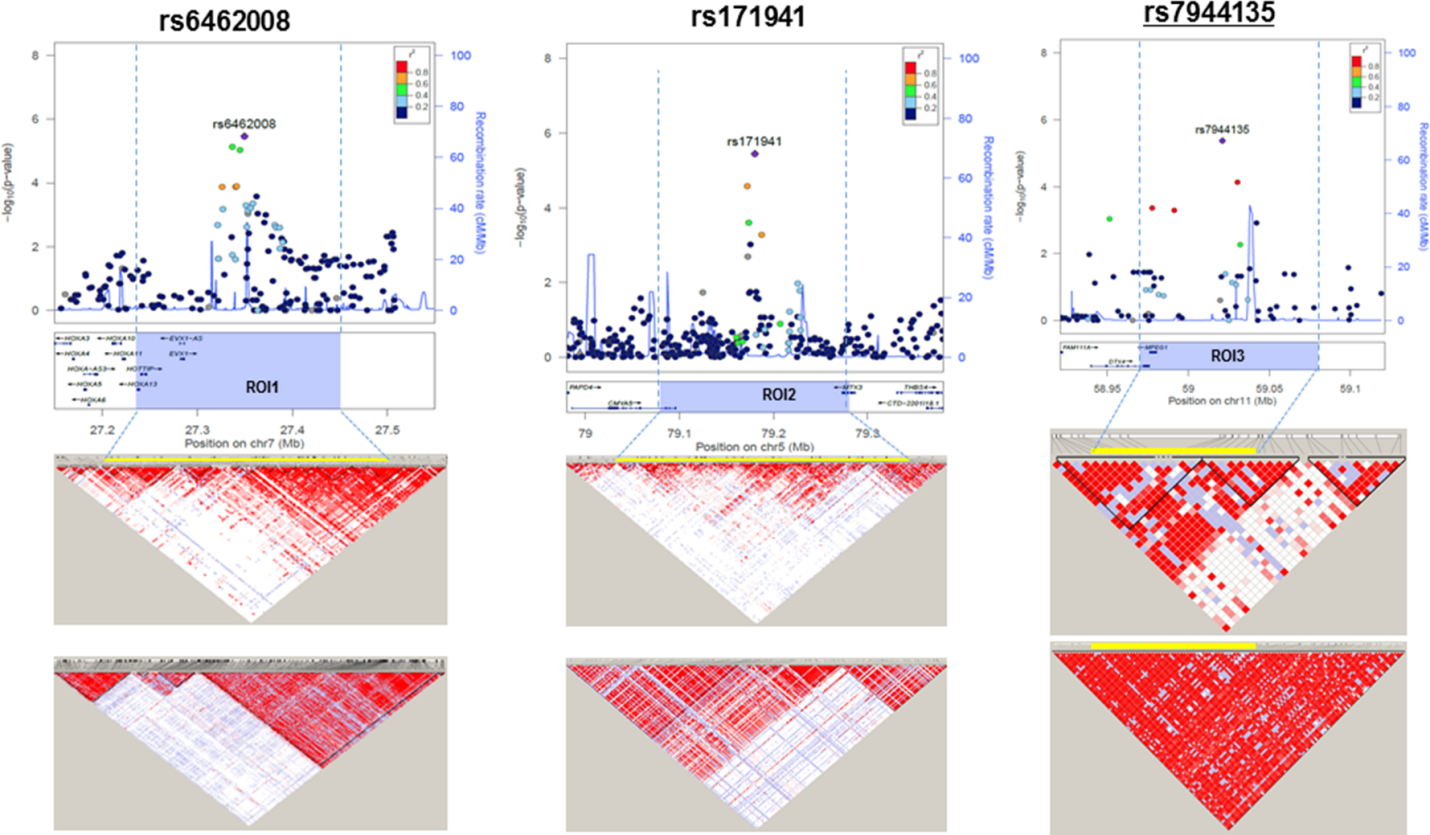
Three regions of interest (ROIs) for susceptibility loci of HBsAg seroclearance. Three ROIs at 7p15.2, 5q14.1, and 11q12.1 are shown using LocusZoom (*top*). For the SNPs in the ROI, −log10 (*P* value) are plotted, and the significant SNPs are indicated. The section of the ROI is indicated with yellow bars. The recombination rates expressed in centimorgans (cM) per Mb (NCBI Build GRCh37) are also shown. The linkage disequilibrium values of the SNPs in the ROIs plotted using Haploview are shown (*bottom*). *D*′-based LD maps are shown for the two different data sets of our Korean cohort (KR) and the public data of the Beijing Han Chinese and Tokyo Japanese cohorts form 1000 Genomes Project data. The *D*′ scores for each paired SNP are indicated using different colors. As the LD increases, the color of the diamond becomes closer to red. The results show that both cohorts have significant LD among the SNPs within ROI. ROI1 and 2 was excluded for further analyses due to low LD (*r*^*2*^ < 0.5).

Next, we determined the ROIs that had at least one SNP with *P*<5.0×10^−6^ and more than two susceptibility-associated SNPs (*P* < 5.0×10^−4^) among the surrounding SNPs within 50 kb. This analysis revealed three ROIs based on the three most significantly associated SNPs (*P* < 5.0×10^−6^) at 7p15.2 (ROI1), 5q14.1 (ROI2), and 11q12.1 (ROI3) (Fig 2). Among these, only ROI3 was valid due to its high linkage disequilibrium (LD) (*r*^*2*^ >0.8) compared with other two ROIs (*r*^*2*^ <0.5).

The rs7944135 at ROI3 was located in an intergenic region at 11q12.1. This ROI included loci for rs3944255 (*P* = 7.11×10^−5^) in an intergenic region and rs5029315 (*P* = 4.29×10^−4^) within the 3’ untranslated region of *MPEG1* (Table 2). Other representative SNPs, i.e., rs7113936 and rs656163 (*P* < 10^−3^) were also included.

**Table 2:** ROI3 enriched with significant SNPs for HBsAg seroclearance in CHB patients.

	SNP	Allele	Position	Gene^a^	MAF		OR	95% CI	*P*-value
					Case	Control			
**ROI3**	rs 5029315	T	Chr11: 58977768	MPEG1	0.349	0.2	2.46	1.49–4.06	4.29×10^−4^
	**rs** **7944135**	**A**	**Chr11:** **59020987**	**DTX4, MPEG1**	**0.31**	**0.125**	**4.16**	**2.27**–**7.63**	**4.17**×**10**^**−6**^
	rs 3944255	T	Chr11: 59030285	DTX4, MPEG1	0.29	0.14	3.16	1.79–5.58	7.11×10^−5^

ROI, region of interest; MAF, minor allele frequency; OR, odds ratio; CI, confidence interval.

a: The flanking genes were located ± 150 kb of each SNP.

ROI3 was located within high LD blocks (*r*^*2*^ = 0.75) showing a strong LD (Fig 2). Moreover, ROI3 showed a high LD in an independent cohort study from the Asian population (Beijing Han Chinese and Tokyo Japanese) in phase 3 of the 1000 Genomes Project [27]. Thus, we suggest that the SNPs in this ROI are highly linked with one another, are heritable, and have a functional significance.

In addition, we further sought to determine whether the haplotype construction using the SNPs in the ROI can improve the statistical significance of the associations with the SNPs. As shown in Table 3, we found that the haplotypes of “T-A” (rs5029315–rs7944135) in ROI3 showed more significant associations (*P* = 3.45×10^−6^, OR = 4.22) than the individual SNPs (rs5029315, *P* = 4.29×10^−4^; rs7944135, *P* = 4.17×10^−6^; rs3944255, *P* = 7.11×10^−5^).

**Table 3:** Haplotype analysis in the ROI for HBsAg seroclearance in patients with CHB.

CHR	BP1	BP2	SNP1	SNP2	SNP3	Haplotype	OR	*P*-value
11	58977768	59020987	rs5029315	rs7944135		TA	4.22	3.45×10^−6^
11	58977768	59030285	rs5029315	rs7944135	rs3944255	TAT	4.48	4.74×10^−6^
11	59020987	59030285	rs7944135	rs3944255		AT	4.46	4.77×10^−6^

The three most significantly associated SNPs within ROI (rs5029315, rs7944135, and rs3944255).

ROI, region of interest; CHB, chronic hepatitis B; CHR, chromosome; BP, base pair; SNP, single nucleotide polymorphism; OR, odds ratio.

### Functional association of the significant SNPs

**Fig 3:**
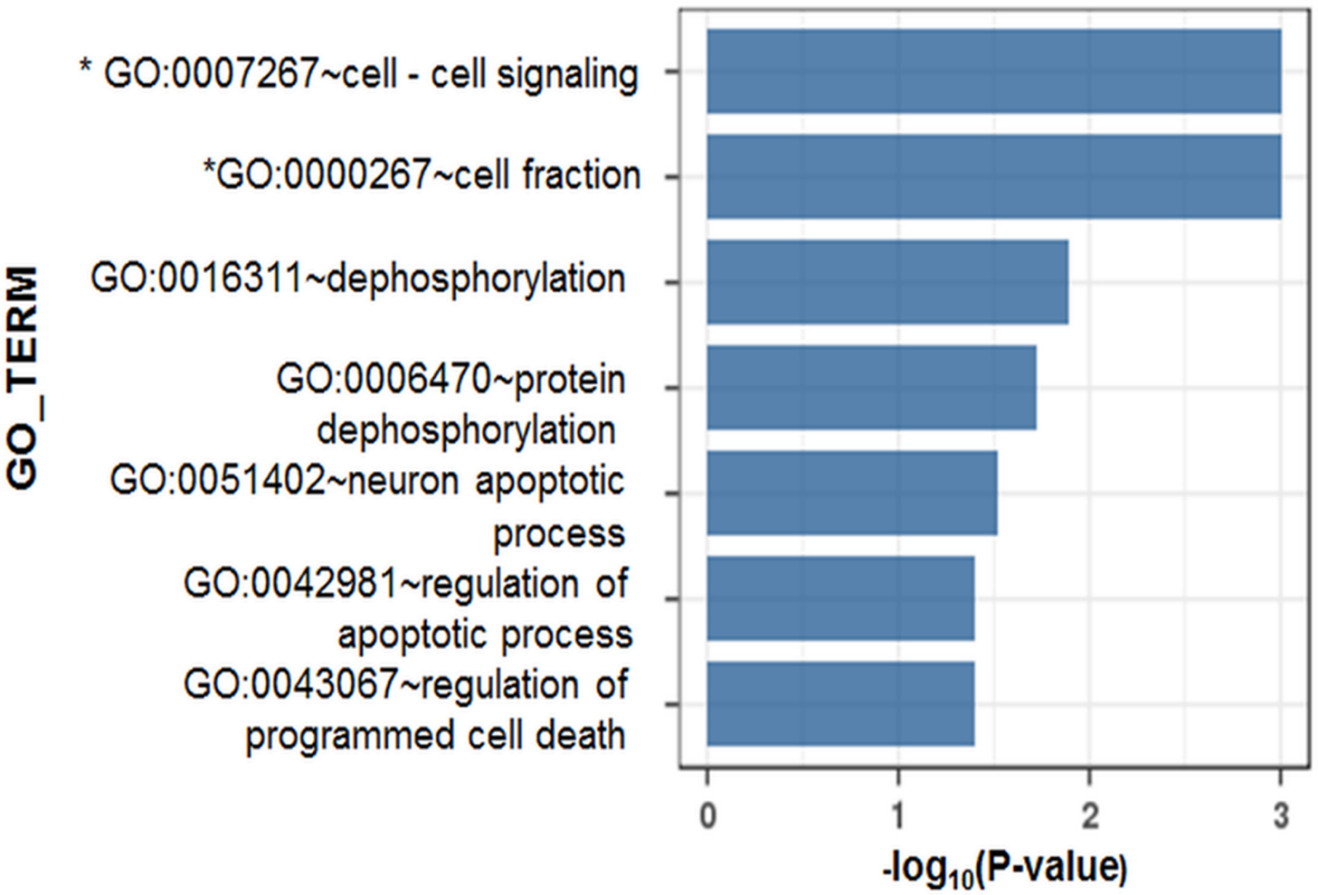
Gene-set enrichment study of the significantly associated SNPs. The functional enrichment among the flanking genes of identified susceptibility-associated SNPs (*P* < 5.0×10^−4)^ was estimated by gene ontology and pathway analyses implemented using DAVID and i-GSEA4GWAS softwares, respectively. The significance of the susceptibility-associated SNPs was estimated with a cutoff of *P* < 5.0×10^−4^. * *P*-values obtained from the pathway analysis are indicated.

Next, we performed gene ontology analysis to determine whether the associated SNPs exhibit a functional enrichment. By inputting the identities of the genes harboring significant SNPs with *P* < 5×10^−4^ to the web-based software DAVID and i-GSEA4GWAS, we found significant functional enrichment in seven gene ontologies (Fig 3). Of these, in particular, the pathways of cell-cell signaling (*P* < 0.001, false discovery rate [FDR] = 0.028) and cell fraction (*P* < 0.001, FDR = 0.059) had highly enriched associations with the identified susceptibility-associated SNPs with an FDR cutoff < 0.2. Among the genes assigned to the cell-cell signaling gene ontology term in this study, C-X-C-chemokine ligand 13 (*CXCL13)* had the most significant flanking SNPs (rs11931577, *P* = 9.85×10^−5^, OR 0.27, 95% CI = 0.14–0.52). These genes have been noted to play important roles in the process of HBV infection.

**Fig 4:**
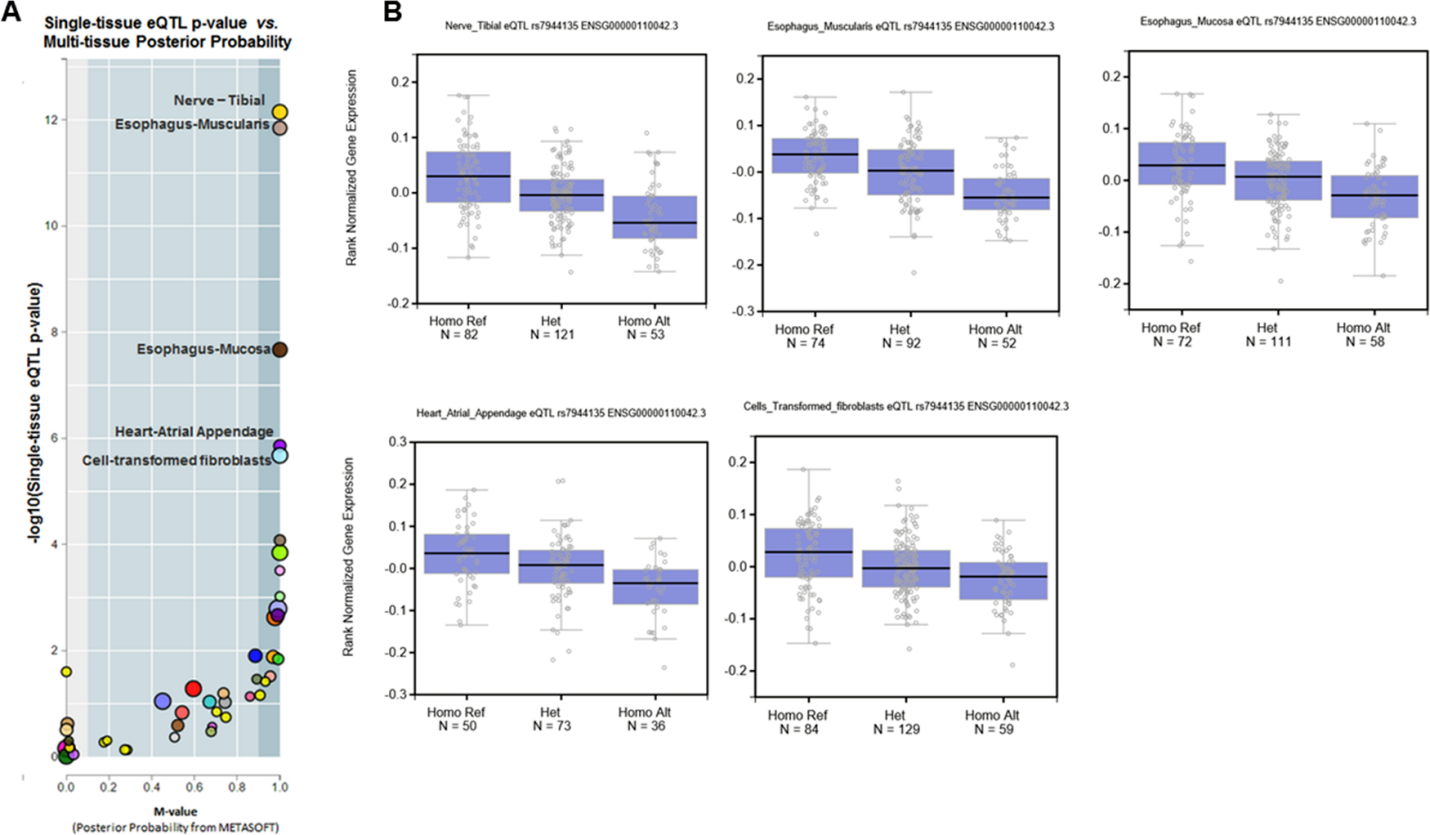
Genotype-tissue expression analysis of rs7944135. **A.** A *cis*-expression quantitative trait locus analysis and multi-tissue posterior probability for rs7944135 and *DTX4* are shown using the GTEx portal database. The five tissues with the highest statistical significance in the *cis*-expression quantitative trait locus analysis (*P*-value < 10^−5^) are indicated. This means that the expression level of *DTX4* significantly differs according to the allele of rs7944135 in those five tissues. **B.** The graphs for expression level of *DTX4* according to the genotypes of rs7944135 in five tissues. With the alteration to A, minor allele, of rs7944135, that was associated with acquisition of HBsAg seroclearance, the expression of *DTX4* is decreased.

To further elaborate the functional significance of the SNPs, we performed a cis-expression quantitative trait loci (QTL) analysis to evaluate the associations of the SNPs with the expression levels of cis-mapped genes. This analysis is based on the correlation between genotype and tissue-specific RNA levels of the target gene. When we examined rs7944135 using the web-based QTL analysis tool GTEX portal (http://www.gtexportal.org), we found that the polymorphism of rs7944135 was significantly associated with the gene expression of the flanking gene *DTX4* in at least five cells or tissue types (nerve, esophagus mucosa and muscularis, fibroblast, and heart, *P* < 1.0×10^−5^; Fig 4). Along with GG, GA, and AA genotypes of rs7944135, the DTX4 expression significantly reduced. Those genotypes were identified in 46/46/8 in case group and 75/25/0 in control group. This result may support the functional relevance of rs7944135 and its flanking gene *DTX4* in the HBsAg seroclearance of patients with CHB.

### Subgroup GWAS regarding HBsAg seroclearance

We performed subgroup analysis to investigate the SNPs associated with HBsAg seroclearance in the subjects who had received antiviral treatment, and further sought the SNPs associated with HBsAg seroconversion, i.e. acquisition of anti-HBs within the case group who had accomplished HBsAg seroclearance. The results are presented in S4 Table. The top 30 SNPs associated with HBsAg seroclearance, except rs171941, rs2153442, and rs4748035, did not overlap between the whole study population and the subpopulation given antiviral treatment, although most of the associated SNPs in each group still maintained statistical significance (P<0.05) in the other group. However, the SNPs associated with the HBsAg seroconversion were completely distinct from those associated with HBsAg seroclearance.

## Discussion

Almost all healthy adults who are not immunosuppressed will rapidly recover from HBV infection following a short-term acute phase. Those rare people who progress to chronicity with adulthood HBV infection might have some genetic defects in the process of clearing HBV. On the other hand, people infected with HBV in infancy or early childhood almost always progress to chronic infections probably because of immature immunity against HBV in that age [25], and recovery from HBV infection in these patients is very rare. Thus, genetic factors associated with HBsAg seroclearance in CHB patients who had been infected in childhood are likely to differ from those determining the chronicization of adulthood HBV infections. In this respect, it is somewhat difficult to define what previous GWASs really meant by their results, since those studies did not exactly clarify the onset time of HBV infection for both patients with and without HBsAg. In all probability, however, the findings of previous GWASs mainly appear to represent genetic markers associated with the chronicization of adult HBV infection rather than viral remission in CHB patients infected in early childhood, for most of previous GWASs were conducted in East Asia, and almost all the participants assigned in the control group without HBsAg are presumed to have recovered from acute phase of adult-onset HBV infection.

To the best of our knowledge, the present study is the first GWAS that attempted to elucidate the genetic factors associated with recovery from chronic HBV infection. To overcome the limitation of having a relatively small number of participants, we performed extreme phenotype GWAS, employing the two groups of patients at opposite poles in HBsAg serodynamics; the subjects that experienced HBsAg seroclearance at < 60 years of age and those who exhibited high serum levels of HBsAg even at ≥ 60 years of age. Furthermore, all the subjects had been confirmed to have HBV infection for a long duration. In addition, this study has the strength of excluding impacts according to heterogeneous HBV genotypes because almost all CHB cases in Korea are infected by HBV genotype C [14].

Most clinical characteristics of the case and control groups in the present study appear to reflect the phenomena occurring as the consequence of different natural history of viral infection, rather than the ones causally related. The serum level of HBV DNA was higher, and anti-HBe seropositivity was lower in subjects showing persistently high level of HBsAg (the control group) compared with those subjects showing HBsAg seroclearance (the case group). HBsAg carriers, who had shown abnormal serum aminotransferase levels during the initial visit, required antiviral treatment in the majority of cases because of persisting viremia and signs of hepatic inflammation. Notably, the treatment periods were not significantly different between the case and control groups, suggesting that antiviral agents did not significantly influence HBsAg seroclearance.

Many studies indicated that male subjects are more susceptible to aggravation of chronic hepatitis B than female subjects because of their sex hormones [28,29], which might suggest strong immune response in males. In similar context, the male predominance in HBsAg-serocleared patients in the present study might suggest the potential impact of gender on HBV elimination in CHB patients in the endemic region of vertical HBV transmission. Comparable findings have been reported in two cohorts in Taiwan and Korea [5,30].

Apart from gender, some SNPs (50 SNPs with *P*-value of <10^−4^) were independently and potentially associated with HBsAg seroclearance in multivariate analysis. Furthermore, the three most significantly associated SNPs (*P* <5.0×10^−6^) and one SNP elicited from web-based functional analysis in the present study have not been revealed to be associated with chronicity of HBV infection in previous GWAS. Although the flanking genes near these susceptibility loci were not reported to be specifically associated with the seroclearance of HBsAg, several studies have shown their associations with HBV infection.

*HOXA13*, which is a flanking gene of rs6462008 and rs6462003 (the 1st and 4th most significant SNPs), is related to the control of cell proliferation, and its expression is increased in hepatocellular carcinoma and liver cirrhosis [31]. Moreover, it was reported to be associated with interferon (IFN) expression [32]. Its sequential action through bone morphogenetic protein 2 (BMP2), JunB, B-cell chronic lymphocytic leukemia/lymphoma 3 (BCL3), and interferon regulatory factor 1 (IRF1) ultimately leads to an increase in the expression of IFN-β or interleukin-1β. This pathway represents an alternative mechanism for the induction of inflammatory and apoptotic genes in the absence of the IFN-α/β receptor during viral infection. CHB might occur during an immunosuppressed state that is mediated by several actions of HBV protein X, such as, down-regulation of type I IFN receptor and the inhibition of extracellular IFN-α-mediated signal transduction [33]. Thus, the inflammatory pathway via HOXA13, an alternative mechanism functioning in the absence of type I IFN receptor, may play a crucial role in the elimination of HBV in patients with CHB.

*MTX3*, the flanking gene of rs171941, encodes a mitochondrial outer membrane protein. This molecule may regulate the permeability of the mitochondrial membrane, and its deficiency may allow cells to resist tumor necrosis factor-induced apoptosis [34]. Because HBV protein X also perturbs mitophagy and apoptosis signals to promote the survival of HBV, a change in the expression of *MTX3* may influence the medical course of CHB.

*MPEG1* and *DTX4*, the flanking genes of rs7944135, appear to be biologically relevant because their functions have been associated with viral clearance through the activation of the IFN pathway. MPEG1 was originally identified as a potential marker of mature macrophages [35], and is one of the interferon-induced genes that inhibit HBV replication in transgenic mouse hepatocytes [36]. Taken together, we suggest that it is biologically plausible that MPEG1 could influence the susceptibility to HBsAg seroclearance.

The immune system of host detects intruding pathogens including HBV by Toll-like receptors (TLRs) and retinoic acid-inducible gene I (RIG-I), and triggers immune reactions such as conjugation of TNF receptor-associated factor 3 (TRAF3) and non-canonical IkB kinases (IKKs), including TANK-binding kinase 1 (TBK1) and IKKi. The complex phosphorylates interferon regulatory factor (IRF), resulting in increased activity of the type I interferons and restricted activity of virus. In chronic HBV infection, HBV protein X suppresses this antiviral pathway [37]. Against this suppression, GS-9620, an oral agonist of TLR-7, has been proposed as a candidate for a new antiviral drug for CHB, as recent studies reported the antiviral effect of this drug [38,39]. DTX4 is also involved with this pathway. It induces TBK1 ubiquitination and degradation through conjugation with nucleotide-binding domain, leucine rich containing family, pyrin domain containing 4 (NLRP4) and thereby restricts the induction of type I IFN signaling [40]. Thus, *DTX4* expression can suppress virus-triggered IFN signaling [37,41,42]. Congruently, our cis-expression QTL analyses demonstrated a functional association between rs7944135 and the expression of *DTX4* using the GTEX database. Presence of the minor allele of rs7944135 that was associated with acquisition of HBsAg seroclearance, lead to decrease in *DTX4* expression. Therefore, these results indicate that the allele alteration of rs7944135 could change the expression of *DTX4* and subsequently influence sustenance of HBV and maintenance of serum titer of HBsAg. However, the association was not confirmed in the liver tissue due to the smaller number of samples. Nonetheless, that association is expected to be reproduced for the liver tissue in the future studies because the significant associations were noted for many tissues. Regarding the linkage disequilibrium analyses, to the rs7944135, haplotypes with rs5029315 had a lower *P-*value and a higher OR than those with rs7944135 alone although its *P-*value was lower than that of rs6462008 alone. Nevertheless, the haplotype of rs7944135 with rs5029315 may contribute an additional benefit for the prediction of HBsAg seroclearance in patients with CHB.

*CXCL13*, which is the flanking gene of rs11931577 in the cell-cell signaling pathway, functions to attract B lymphocytes and promote the migration and aggregation. It is expressed in an age-dependent manner in mouse hepatic macrophages and plays an integral role in facilitating an effective immune response against HBV [43]. A high level of CXCL13 contributes to the control of the viral load during treatment with nucleos(t)ide analogues [44]. Its action may also contribute to achieving HBsAg seroclearance.

In addition, subgroup analysis revealed some SNPs that were associated with HBsAg seroclearance in the subjects who received antiviral treatment. In particular, rs171941 was highly associated with HBsAg seroclearance not only in the whole study population but also in the subgroup that received antiviral treatment, suggesting its important association with HBsAg serodynamics during antiviral treatment. Also, many SNPs were found significantly associated with the acquisition of anti-HBs within the subjects who attained HBsAg seroclearance. This indicates that some additional genetic factors might be related to the appearance of anti-HBs after the occurrence of HBsAg seroclearance. However, we did not conduct a further bioinformatics data search for those SNPs, considering the small number of subjects in the subgroups.

In summary, the present study is the first GWAS to investigate the genetic predisposition associated with the functional cure in patients with CHB. Our GWAS analysis revealed three novel susceptibility loci for HBsAg seroclearance in a Korean CHB population, including rs6462008 at 7p15.2, rs171941 at 5q14.1 and rs7944135 at 11q12.1. These SNPs are located adjacent to the *HOXA13*, *MTX3*, *MPEG1* and *DTX4* genes, respectively. In particular, rs7944135 showed a functional relevance with *DTX4* gene expression in web-based bioinformatics analysis. Moreover, another SNP rs11931577 is located near *CXCL13* gene, and modulates the immune response against HBV infection. Further investigations are required for the validation of significant SNPs and the identification of the associations between those SNPs and the functions of flanking genes.

The results of the present study might be helpful for predicting the clinical outcomes of patients with CHB and elucidating the mechanism of viral elimination in chronic HBV infection. In addition, the SNPs and their flanking genes that are suggested to be associated with HBsAg seroclearance might act as targets for new pharmaceutical CHB treatments and represent clinical evidence for considering the discontinuation of antiviral treatment in patients with CHB.

## Supporting information

S1 Fig Quantile–quantile plot of the genome wide association study (GWAS). The distributions of observed p-values did not deviate from the null distribution, which excluded systematic bias due to bad genotyping or population structure. The y-axis is the observed -log10(p) values; the x-axis is the expected -log10(p) values. The genomic control inflation factor (λ) is 0.893.
(PPTX)

S1 Table Clinical features of participants according to presence of family history of HBV infection. Data were presented as number (%), mean ± standard deviation, or median (IQR). ALT, alanine aminotransferase (normal range 5–40 U/L); HBeAg, hepatitis B e antigen; IFN, interferon (including PEGylated-interferon); NA, nucleos(t)ide analogues.
(DOCX)

S2 Table List of significant (*P*< 10^−4^) SNPs associated with HBsAg seroclearance. The significant SNPs (*p* < 1.0ⅹ10^−4^) are presented. SNP, single nucleotide polymorphism; rsID, reference SNP identity; Chr, chromosome; A1, minor allele in the entire cohort; A2, major allele; OR, odds ratio; CI, confidence interval. a: The flanking genes were located ± 150 kb of each SNP. The odds ratio for the minor allele in an additive model. *P*-value was obtained by logistic regression test for the minor allele additive model.
(DOCX)

S3 Table Summary of SNPs that were significantly associated with the persistent HBV infection in previous GWASs. SNP, single nucleotide polymorphism; GWAS, genome-wide association study; rsID, reference SNP identity; A1, minor allele; A2, major allele; chr, chromosome; OR, odds ratio; CI, confidence interval; PMID, pubmed identity. The odds ratio for the minor allele in a additive model. *P*-value was obtained by logistic regression test for the minor allele additive model.
(DOCX)

S4 Table Summary of top 30 SNPs that were significantly associated with the HBsAg seroclearance, HBsAb seroconversion and response to antiviral treatment. The most significant SNPs (up to 30 SNPs) are presented according to each outcome with *P*-values of other outcomes, as well. SNP, single nucleotide polymorphism; rsID, reference SNP identity; Chr, chromosome. a: The flanking genes were located ± 150 kb of each SNP. The odds ratio for the minor allele in an additive model. *P*-value was obtained by logistic regression test for the minor allele additive model.
(XLSX)

## Abbreviations

CHB: chronic hepatitis B
HBsAg: hepatitis B surface antigen
HBV: hepatitis B virus
GWAS: genome-wide association study
HLA: human leukocyte antigen
SNP: single-nucleotide polymorphism
IFN: interferon
