# Supplementary material for: Identification of novel susceptibility loci associated with hepatitis B surface antigen seroclearance in chronic hepatitis B
Source: PLoS One. 2018 Jul 5;13(7):e0199094. doi: 10.1371/journal.pone.0199094 (PMC6033413; doi:10.1371/journal.pone.0199094)
Supplement: S1 Fig — The distributions of observed p-values did not deviate from the null distribution, which excluded systematic bias due to bad genotyping or population structure. The y-axis is the observed -log10(p) values; the x-axis is the expected -log10(p) values. The genomic control inflation factor (λ) is 0.893. (PPTX) [file pone.0199094.s001.pptx]

## Slide 1
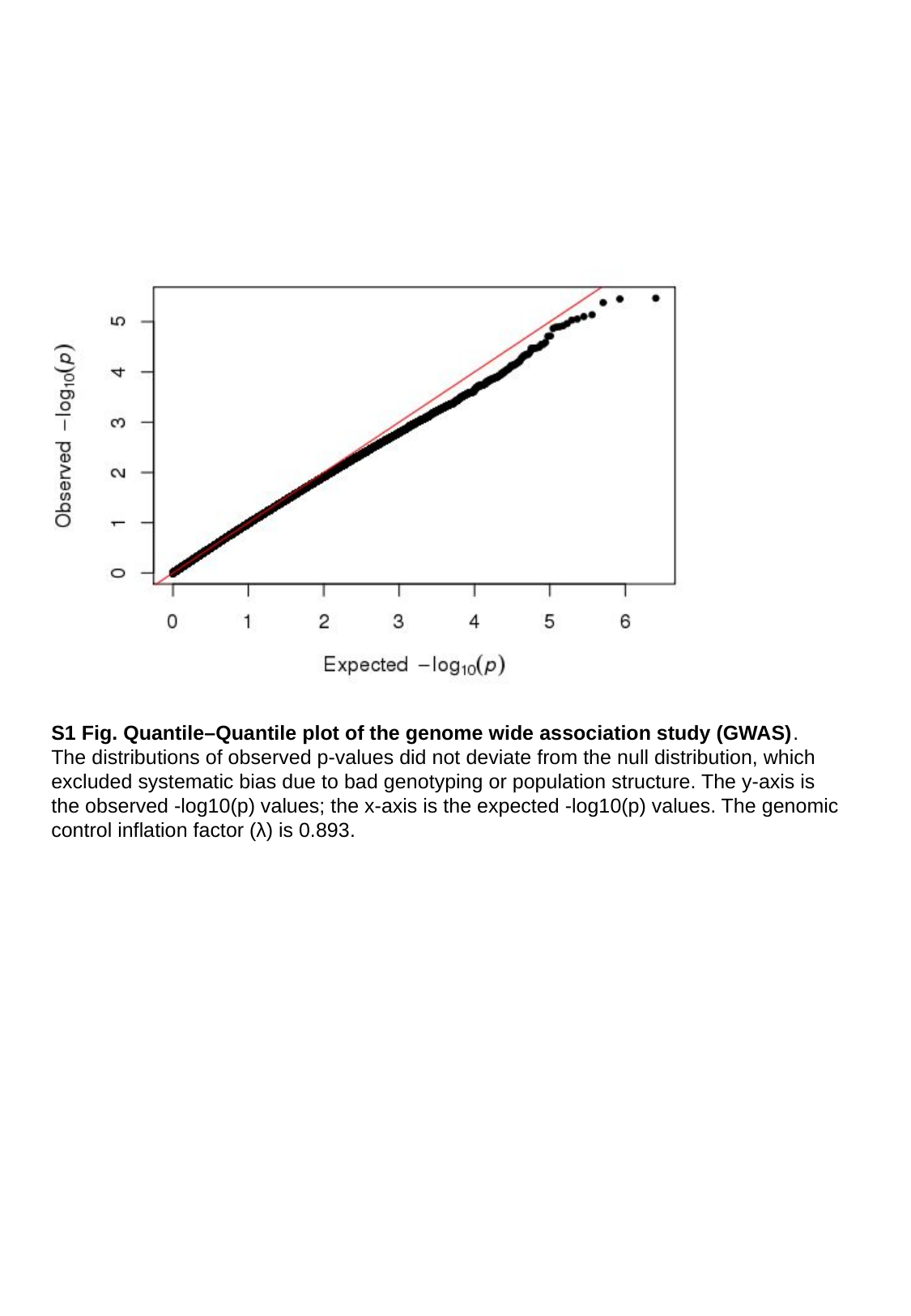

S1 Fig. Quantile–Quantile plot of the genome wide association study (GWAS).
The distributions of observed p-values did not deviate from the null distribution, which excluded systematic bias due to bad genotyping or population structure. The y-axis is the observed -log10(p) values; the x-axis is the expected -log10(p) values. The genomic control inflation factor (λ) is 0.893.
