# Supplementary material for: Identification of novel susceptibility loci associated with hepatitis B surface antigen seroclearance in chronic hepatitis B
Source: PLoS One. 2018 Jul 5;13(7):e0199094. doi: 10.1371/journal.pone.0199094 (PMC6033413; doi:10.1371/journal.pone.0199094)
Supplement: S1 Table — Data were presented as number (%), mean ± standard deviation, or median (IQR). ALT, alanine aminotransferase (normal range 5–40 U/L); HBeAg, hepatitis B e antigen; IFN, interferon (including PEGylated-interferon); NA, nucleos(t)ide analogues. (DOCX) [file pone.0199094.s002.docx]

**S1 Table.** **Clinical features of participants according to presence of family history of HBV infection**

|  | | **Case** | | **Control** | |
| --- | --- | --- | --- | --- | --- |
| **Family history of HBV infection** | | Present  (*n*=70) | Not confirmed (*n*=30) | Present  (*n*=81) | Not confirmed (*n*=19) |
| **Male, *n*** | | 45 (64.3) | 26 (86.7) | 33 (40.7) | 12 (63.2) |
| **Age at initial diagnosis, years** | | 36.0±11.1 | 36.1±12.2 | 45.8±13.0 | 52.4±12.0 |
| **Age at 1^st^ visit, years** | | 43.4±10.6 | 45.1±13.2 | 59.3±7.9 | 62.6±6.8 |
| **Age at last visit, years** | | 60.8±9.1 | 60.1±9.8 | 69.0±5.5 | 70.5±5.2 |
| **Follow-up duration, years** | | 17.40±7.9 | 15.0±8.4 | 9.8±6.1 | 8.0±4.4 |
| **Age at HBsAg seroclearance, years** | | 54.8±8.8 | 54.3±9.5 | - | - |
| **ALT at 1st visit, IU/L** | | 35 (17-71) | 47 (17-108) | 47 (25-99) | 61 (35-127) |
| **ALT at GWAS, IU/L** | | 19 (13-26) | 18 (14-29) | 21 (17-27) | 27 (21-30) |
| **HBeAg (+) at 1^st^ visit, *n*** | | 19 (27.1) | 11 (36.7) | 43 (53.1) | 9 (47.4) |
| **HBeAg (+) at GWAS, *n*** | | 0 | 0 | 12 (14.8) | 1 (5.3) |
| **anti-HBe (+) at 1^st^ visit, *n*** | | 50 (71.4) | 20 (66.7) | 33 (40.7) | 9 (47.4) |
| **anti-HBe (+) at GWAS, *n*** | | 65 (92.9) | 25 (83.3) | 49 (60.5) | 11 (57.9) |
| **HBsAg (+) at 1^st^ visit, *n*** | | 70 | 30 | 81 | 19 |
| **HBsAg (+) at GWAS, *n*** | | 0 | 0 | 81 | 19 |
| **Anti-HBs (+) at GWAS, *n*** | | 47 (67.1) | 19 (63.3) | 0 | 0 |
| **HBV DNA < 20 IU/mL at GWAS, *n*** | | 65 (92.9) | 29 (96.7) | 58 (71.6) | 17 (89.5) |
| **Treatment type** | **Non-treated, *n*** | 50 (71.4) | 19 (63.3) | 13 (16.0) | 3 (15.8) |
|  | **only IFN, *n*** | 5 (7.1) | 2 (6.7) | 0 | 0 |
|  | **only NA, *n*** | 13 (18.6) | 9 (30.0) | 65 (80.2) | 16 (84.2) |
|  | **IFN & NA, *n*** | 2 (2.9) | 0 | 3 (3.7) | 0 |
| **Age of treatment start, years** | | 42.9±11.6 | 43.4±12.2 | 61.0±6.9 | 62.8±5.1 |
| **NA treatment period, years** | | 8.5±3.3 | 5.5±3.6 | 7.9±4.3 | 6.9±3.0 |

Data were presented as number (%), mean ± standard deviation, or median (IQR).

ALT, alanine aminotransferase (normal range 5–40 U/L); HBeAg, hepatitis B e antigen; IFN, interferon (including PEGylated-interferon); NA, nucleos(t)ide analogues.
