# Supplementary material for: Identification of novel susceptibility loci associated with hepatitis B surface antigen seroclearance in chronic hepatitis B
Source: PLoS One. 2018 Jul 5;13(7):e0199094. doi: 10.1371/journal.pone.0199094 (PMC6033413; doi:10.1371/journal.pone.0199094)
Supplement: S2 Table — The significant SNPs (p < 1.0ⅹ10−4) are presented. SNP, single nucleotide polymorphism; rsID, reference SNP identity; Chr, chromosome; A1, minor allele in the entire cohort; A2, major allele; OR, odds ratio; CI, confidence interval. a: The flanking genes were located ± 150 kb of each SNP. The odds ratio for the minor allele in an additive model. P-value was obtained by logistic regression test for the minor allele additive model. (DOCX) [file pone.0199094.s003.docx]

**S2 Table. List of significant (*P*< 10^-4^) SNPs associated with HBsAg seroclearance.**

| **rsID** | **Chr** | **Position** | **Cytoband** | **Gene^a^** | **A1/A2** | **minor allele frequency** | | **OR (95% CI)** | ***P*** |
| --- | --- | --- | --- | --- | --- | --- | --- | --- | --- |
|  |  |  |  |  |  | **Case** | **Control** |  |  |
| rs6462008 | 7 | 27349479 | 7p15.2 | *EVX1, HOXA13* | G/T | 0.37 | 0.58 | 0.34(0.22-0.54) | 3.40x10^-6^ |
| rs171941 | 5 | 79180126 | 5q14.1 | *CMYA5, MTX3* | A/G | 0.38 | 0.19 | 3.69(2.13-6.42) | 3.52x10^-6^ |
| rs7944135 | 11 | 59020987 | 11q12.1 | *DTX4, MPEG1* | A/G | 0.31 | 0.13 | 4.16(2.27-7.63) | 4.17x10^-6^ |
| rs6462003 | 7 | 27336745 | 7p15.2 | *HOXA13* | A/G | 0.35 | 0.57 | 0.35(0.22-0.55) | 7.26x10^-6^ |
| rs199869387 | 2 | 59708649 | 2p16.1 | *-* | A/G | 0.56 | 0.35 | 2.97(1.84-4.79) | 7.89x10^-6^ |
| rs12463513 | 2 | 59644842 | 2p16.1 | *-* | A/G | 0.57 | 0.34 | 2.67(1.73-4.11) | 8.85x10^-6^ |
| rs6947275 | 7 | 27344799 | 7p15.2 | *HOTTIP,*  *EVX1* | C/G | 0.36 | 0.57 | 0.35(0.22-0.56) | 9.27x10^-6^ |
| rs7230406 | 18 | 61863854 | 18q22.1 | *LOC284294* | A/G | 0.11 | 0.30 | 0.27(0.15-0.48) | 1.09x10^-5^ |
| rs13018470 | 2 | 59694749 | 2p16.1 | *-* | T/C | 0.56 | 0.36 | 2.92(1.81-4.72) | 1.21x10^-5^ |
| rs7945342 | 11 | 5543705 | 11p15.4 | *OR51B5* | T/C | 0.29 | 0.52 | 0.34(0.21-0.55) | 1.26x10^-5^ |
| rs2192611 | 2 | 59695598 | 2p16.1 | *-* | A/C | 0.56 | 0.35 | 2.91(1.80-4.70) | 1.28x10^-5^ |
| rs1809862 | 11 | 5537780 | 11p15.4 | *UBQLNL* | A/C | 0.29 | 0.51 | 0.34(0.21-0.55) | 1.36x10^-5^ |
| rs4377248 | 18 | 61862921 | 18q22.1 | *LOC284294* | A/C | 0.11 | 0.29 | 0.28(0.15-0.50) | 1.92x10^-5^ |
| rs62439524 | 7 | 2684070 | 7p22.3 | *TTYH3* | G/C | 0.57 | 0.39 | 2.94(1.79-4.81) | 1.95x10^-5^ |
| rs265005 | 5 | 79172136 | 5q14.1 | *CMYA5* | C/T | 0.33 | 0.16 | 3.24(1.87-5.60) | 2.57x10^-5^ |
| rs12464531 | 2 | 59673530 | 2p16.1 | *-* | T/C | 0.56 | 0.37 | 2.76(1.72-4.44) | 2.82x10^-5^ |
| rs2215905 | 2 | 59678265 | 2p16.1 | *-* | C/T | 0.56 | 0.37 | 2.76(1.72-4.44) | 2.82x10^-5^ |
| rs11891860 | 2 | 59667692 | 2p16.1 | *-* | A/G | 0.34 | 0.55 | 0.40(0.26-0.61) | 3.20x10^-5^ |
| rs1558599 | 2 | 59663978 | 2p16.1 | *-* | T/C | 0.34 | 0.56 | 0.40(0.26-0.62) | 3.27x10^-5^ |
| rs887941 | 2 | 59686958 | 2p16.1 | *-* | C/T | 0.56 | 0.37 | 2.69(1.69-4.30) | 3.34x10^-5^ |
| rs2017434 | 11 | 5536852 | 11p15.4 | *UBQLNL* | G/A | 0.32 | 0.53 | 0.37(0.23-0.59) | 3.38x10^-5^ |
| rs872751 | 11 | 5537045 | 11p15.4 | *UBQLNL* | G/T | 0.32 | 0.53 | 0.37(0.23-0.59) | 3.38x10^-5^ |
| rs2047456 | 11 | 5537161 | 11p15.4 | *UBQLNL* | C/T | 0.32 | 0.53 | 0.37(0.23-0.59) | 3.38x10^-5^ |
| rs12593003 | 15 | 69896675 | 15q23 | *DRAIC* | A/G | 0.15 | 0.35 | 0.32(0.18-0.55) | 4.07x10^-5^ |
| rs10769023 | 11 | 5536415 | 11p15.4 | *UBQLNL* | T/C | 0.32 | 0.53 | 0.37(0.23-0.60) | 4.44x10^-5^ |
| rs17584600 | 2 | 59661521 | 2p16.1 | *-* | A/G | 0.35 | 0.57 | 0.41(0.26-0.63) | 4.49x10^-5^ |
| rs8037510 | 15 | 86797384 | 15q25.3 | *AGBL1* | A/G | 0.15 | 0.32 | 0.32(0.18-0.55) | 4.49x10^-5^ |
| rs2153442 | 10 | 13511130 | 10p13 | *BEND7* | G/A | 0.32 | 0.13 | 3.15(1.81-5.47) | 4.79x10^-5^ |
| rs4748035 | 10 | 13511986 | 10p13 | *BEND7* | T/C | 0.32 | 0.13 | 3.15(1.81-5.47) | 4.79x10^-5^ |
| rs12620748 | 2 | 6866987 | 2p25.2 | *LINC01246* | T/C | 0.35 | 0.19 | 2.93(1.74-4.94) | 5.18x10^-5^ |
| rs6737829 | 2 | 59669528 | 2p16.1 | *-* | C/T | 0.39 | 0.22 | 2.79(1.70-4.58) | 5.34x10^-5^ |
| rs13033709 | 2 | 49490690 | 2p16.3 | *-* | A/G | 0.07 | 0.22 | 0.24(0.12-0.48) | 5.92x10^-5^ |
| rs12051751 | 17 | 16501887 | 17p11.2 | *CCDC144A, ZNF287* | A/C | 0.58 | 0.37 | 2.44(1.58-3.77) | 6.31x10^-5^ |
| rs2173091 | 15 | 86797399 | 15q25.3 | *AGBL1* | G/A | 0.17 | 0.35 | 0.35(0.21-0.58) | 6.34x10^-5^ |
| rs1546196 | 12 | 25085390 | 12p12.1 | *BCAT1* | T/C | 0.44 | 0.25 | 2.66(0.25-1.64) | 6.60x10^-5^ |
| rs1840440 | 18 | 23257186 | 18q11.2 | *-* | C/T | 0.37 | 0.57 | 0.37(0.23-0.60) | 6.86x10^-5^ |
| rs6840435 | 4 | 5813695 | 4p16.2 | *EVC* | C/T | 0.30 | 0.46 | 0.38(0.23-0.61) | 7.07x10^-5^ |
| rs3944255 | 11 | 59030285 | 11q12.1 | *DTX4, MPEG1* | T/C | 0.29 | 0.14 | 3.16(1.79-5.58) | 7.11x10^-5^ |
| rs9296504 | 6 | 46629505 | 6p12.3 | *SLC25A27* | C/T | 0.38 | 0.57 | 0.42(0.28-0.65) | 7.44x10^-5^ |
| rs9381469 | 6 | 46630068 | 6p12.3 | *SLC25A27* | A/G | 0.38 | 0.57 | 0.42(0.28-0.65) | 7.44x10^-5^ |
| rs9911677 | 17 | 16537397 | 17p11.2 | *ZNF624* | T/C | 0.59 | 0.38 | 2.42(1.56-3.75) | 7.44x10^-5^ |
| rs12151705 | 2 | 59738664 | 2p16.1 | *-* | G/T | 0.39 | 0.22 | 2.72(1.66-4.47) | 7.46x10^-5^ |
| rs7644288 | 3 | 172328281 | 3q26.31 | *-* | A/T | 0.20 | 0.07 | 4.06(2.03-8.16) | 7.96x10^-5^ |
| rs10508462 | 10 | 13519743 | 10p13 | *BEND7* | G/T | 0.32 | 0.14 | 2.99(1.73-5.16) | 8.36x10^-5^ |
| rs2191314 | 7 | 20355658 | 7p21.1 | *MACC1* | T/C | 0.48 | 0.30 | 2.61(1.62-4.22) | 8.70x10^-5^ |
| rs13382813 | 2 | 6852941 | 2p25.2 | *LINC01246* | A/G | 0.32 | 0.17 | 2.95(1.72-5.06) | 8.76x10^-5^ |
| rs10838245 | 11 | 5535529 | 11p15.4 | *OR51B5* | T/G | 0.32 | 0.52 | 0.38(0.24-0.62) | 8.92x10^-5^ |
| rs1482088 | 4 | 86559173 | 4q21.23 | *ARHGAP24* | C/A | 0.57 | 0.39 | 2.44(1.56-3.82) | 9.18x10^-5^ |
| rs7073919 | 10 | 98926961 | 10q24.1 | *SLIT1* | G/A | 0.10 | 0.24 | 0.28(0.14-0.53) | 9.30x10^-5^ |
| rs6749972 | 2 | 59651584 | 2p16.1 | *-* | T/C | 0.36 | 0.56 | 0.42(0.28-0.65) | 9.56x10^-5^ |
| rs11931577 | 4 | 78335569 | 4q21.1 | *CXCL13* | C/T | 0.10 | 0.23 | 0.27(0.14-0.52) | 9.85x10^-5^ |
| rs6790457 | 3 | 156374086 | 3q25.31 | *LINC00886* | A/G | 0.21 | 0.08 | 3.96(1.98-7.92) | 9.94x10^-5^ |

The significant SNPs (p < 1.0ⅹ10-4) are presented.

SNP, single nucleotide polymorphism; rsID, reference SNP identity; Chr, chromosome; A1, minor allele in the entire cohort; A2, major allele; OR, odds ratio; CI, confidence interval.

a: The flanking genes were located ± 150 kb of each SNP.

The odds ratio for the minor allele in an additive model. *P*-value was obtained by logistic regression test for the minor allele additive model.
