# Supplementary material for: Identification of novel susceptibility loci associated with hepatitis B surface antigen seroclearance in chronic hepatitis B
Source: PLoS One. 2018 Jul 5;13(7):e0199094. doi: 10.1371/journal.pone.0199094 (PMC6033413; doi:10.1371/journal.pone.0199094)
Supplement: S3 Table — SNP, single nucleotide polymorphism; GWAS, genome-wide association study; rsID, reference SNP identity; A1, minor allele; A2, major allele; chr, chromosome; OR, odds ratio; CI, confidence interval; PMID, pubmed identity. The odds ratio for the minor allele in a additive model. P-value was obtained by logistic regression test for the minor allele additive model. (DOCX) [file pone.0199094.s004.docx]

**S3 Table. Summary of SNPs that were significantly associated with the persistent HBV infection in previous GWASs.**

| **RsIDs** | **Chromosome positions** | **Genes** | **A1/A2** | **OR  (95% CI)** | ***P*** | **In our GWAS data** | | | | **PMID**  **number** |
| --- | --- | --- | --- | --- | --- | --- | --- | --- | --- | --- |
|  |  |  |  |  |  | **Minor allele frequency** | | **OR (95% CI)** | ***P*** |  |
|  |  |  |  |  |  | **Cases** | **Controls** |  |  |  |
| rs1419881 | *chr6:31130593* | *TCF19* | C/T | 0.73  (0.66-0.81) | 1.3x10^-18^ | 0.28 | 0.38 | 0.6  (0.39-0.94) | 0.024 | 23760081 |
| rs3130542 | *chr6:31232111* | *HLA-C* | A/G | 0.75  (0.69-0.81) | 9.5x10^-14^ | 0.12 | 0.16 | 0.67  (0.36-1.24) | 0.201 | 24162738 |
| rs652888 | *chr6:31851234* | *EHMT2* | C/T | 1.38  (1.22-1.57) | 7.1x10^-13^ | 0.16 | 0.23 | 0.62  (0.37-1.07) | 0.085 | 23760081 |
| rs12614 | *chr6:31914179* | *CFB* | T/C | 0.53  (0.48-0.59) | 1.3x10^-34^ | 0.04 | 0.05 | 0.89  (0.31-2.6) | 0.831 | 25802187 |
| rs2856718 | *chr6:32670255* | *HLA-DQA2,*  *HLA-DQB1* | G/A | 0.64  (0.53-0.78) | 4.0x10^-37^ | 0.46 | 0.48 | 0.90  (0.59-1.37) | 0.621 | 21750111 |
| rs7453920 | *chr6:32730012* | *HLA-DQB2* | A/G | 1.81  (1.62-2.01) | 6.0x10^-28^ | 0.08 | 0.11 | 0.72  (0.35-1.5) | 0.386 | 21750111 |
| rs2395309 | *chr6:33026246* | *HLA-DPA1* | A/G | 1.81  (1.62-2.01) | 1.6x10^-05^ | 0.27 | 0.34 | 0.7  (0.45-1.07) | 0.101 | 19349983 |
| rs3077 | *chr6:33033022* | *HLA-DPA1* | T/C | 0.56  (0.51-0.61) | 2.3x10^-38^ | 0.27 | 0.34 | 0.7  (0.45-1.07) | 0.101 | 19349983 |
| rs2301220 | *chr6:33038766* | *HLA-DPA1* | G/A | NA | 4.5x10^-06^ | 0.26 | 0.33 | 0.69  (0.45-1.08) | 0.103 | 19349983 |
| rs9277341 | *chr6:33039625* | *HLA-DPA1* | T/C | NA | 5.1x10^-3^ | 0.09 | 0.13 | 0.73  (0.39-1.39) | 0.341 | 19349983 |
| rs3135021 | *chr6:33045558* | *HLA-DPB1* | A/G | NA | 3.5x10^-3^ | ND | ND | ND | ND | 19349983 |
| rs9277535 | *chr6:33054861* | *HLA-DPB1* | A/G | 0.57  (0.52-0.62) | 6.3x10^-39^ | 0.33 | 0.38 | 0.78  (0.52-1.17) | 0.228 | 19349983 |
| rs10484569 | *chr6:33058952* | *HLA-DPB1* | A/G | NA | 1.6x10^-3^ | 0.51 | 0.42 | 1.57  (1.03-2.40) | 0.035 | 19349983 |
| rs3128917 | *chr6:33059996* | *HLA-DPB1* | T/G | NA | 1.6x10^-4^ | 0.41 | 0.46 | 0.79  (0.53-1.18) | 0.256 | 19349983 |
| rs2281388 | *chr6:33060118* | *HLA-DPB1* | T/C | NA | 2.6x10^-3^ | 0.49 | 0.41 | 1.5  (0.98-2.32) | 0.06 | 19349983 |
| rs3117222 | *chr6:33060949* | *HLA-DPB1* | G/A | NA | 2.1x10^-4^ | 0.41 | 0.46 | 0.8  (0.54-1.20) | 0.285 | 19349983 |
| rs9380343 | *chr6:33079166* | *HLA-DPB1* | T/C | NA | 3.2 x10^-4^ | 0.49 | 0.43 | 1.51  (0.98-2.32) | 0.059 | 19349983 |
| rs421446 | *chr6:33174783* | *COL11A2* | T/C | 0.73  (0.62–0.87) | 5.0 x10^-4^ | 0.32 | 0.28 | 1.08  (0.7-1.67) | 0.732 | 23508906 |
| rs107822 | *chr6:33175575* | *COL11A2* | G/A | 0.79  (0.67–0.94) | 8.0 x10^-3^ | 0.36 | 0.38 | 0.88  (0.6-1.43) | 0.54 | 23508906 |
| rs7000921 | *chr8:20393206* | *INTS10* | T/C | 0.78  (0.73-0.84) | 3.2x10^-12^ | 0.24 | 0.26 | 0.83  (0.52-1.33) | 0.437 | 27244555 |
| rs11991803 | *chr8:20400460* | *INTS10* | A/G | NA | NA | ND | ND | ND | ND | 27244555 |
| rs4922214 | *chr8:20388526* | *INTS10* | C/T | NA | NA | 0.17 | 0.19 | 0.81  (0.46-1.41) | 0.454 | 27244555 |
| rs1883832 | *chr20:44746982* | *CD40* | T/C | 1.19  (1.14-1.25) | 3.0 x10^-15^ | 0.36 | 0.39 | 0.8  (0.52-1.22) | 0.303 | 25802187 |
| rs4821116 | *chr22:21973319* | *UBE2L3* | A/G | 1.22  (1.15-1.30) | 1.7 x10^-12^ | 0.3 | 0.33 | 0.84  (0.55-1.27) | 0.397 | 24162738 |

SNP, single nucleotide polymorphism; GWAS, genome-wide association study; rsID, reference SNP identity; A1, minor allele; A2, major allele; chr, chromosome; OR, odds ratio; CI, confidence interval; PMID, pubmed identity.

The odds ratio for the minor allele in an additive model. *P*-value was obtained by logistic regression test for the minor allele additive model.
